# Association of maternal prepregnancy weight and early childhood weight with obesity in adolescence: A population‐based longitudinal cohort study in Japan

**DOI:** 10.1111/ijpo.12597

**Published:** 2020-01-07

**Authors:** Satomi Yoshida, Takeshi Kimura, Masahiro Noda, Masato Takeuchi, Koji Kawakami

**Affiliations:** ^1^ Department of Pharmacoepidemiology, Graduate School of Medicine and Public Health Kyoto University Kyoto Japan

**Keywords:** Birth weight, early childhood, epidemiology, health check‐up, maternal factors, obesity in adolescence

## Abstract

**Background:**

The impact of birth weight and obesity in early childhood on obesity in adolescence remains unclear.

**Objectives:**

To examine the association of overweight/obesity at age 15 years with birth weight, overweight/obesity in early childhood and overweight/obesity in mothers.

**Methods:**

This population‐based retrospective cohort study used early childhood and school age health check‐up data of 1581 children in Japan, followed‐up until age 15 years. Generalized estimation equation analyses were used to investigate the association of overweight/obesity at age 15 years with low/high birth weight, overweight/obesity in 3 years of age and overweight/obesity in mothers. The cutoff points for all variables were defined by international criteria.

**Results:**

Of 1581 mother‐child pairs, 130 (8.2%) children had low birth weight, while 93 (5.9%) and 167 (10.6%) were overweight/obese at age 3 and 15 years, respectively. Overweight/obesity at age 3 years and overweight/obesity in mothers were associated with overweight/obesity at age 15 years (adjusted odds ratio [aOR], 4.26; 95% confidence interval [CI]: 2.51–7.25 and (aOR, 2.46; 95% CI: 1.41–4.30). No association between low birth weight and overweight/obesity at age 15 years was observed.

**Conclusions:**

Overweight/obesity in mothers and overweight/obesity at 3 years of age, but not birth weight, were associated with overweight/obesity at age 15 years.

**Abbreviations**
aOR: adjusted odds ratio
BMI: body mass index
CI: confidence interval

## 1 INTRODUCTION

The increasing prevalence of obesity among children is a major health problem worldwide.^1^ Obesity in childhood can increase the risk of premature onset of illnesses, including diabetes and cardiovascular diseases, and increase the risk of death in adulthood.^2^, ^3^, ^4^ Though high birth weight is associated with increased risk of obesity in childhood, the association between low birth weight and the risk of obesity in childhood is controversial.^5^, ^6^, ^7^ One meta‐analysis reported that birth weight over 4000 g was associated with increased risk of obesity in later life, compared either with those weighing ≤4000 g or with the reference group of normal birth weight (between 2500 to 4000 g). However, no clear difference (increase or decrease) was reported in the prevalence of obesity in children with low birth weight (<2500 g) compared with either birth weight >2500 g or with the reference group of normal birth weight (between 2500 to 4000 g).^6^

Recent accumulating evidence suggests that obesity in early childhood is also an important predictor of obesity in adolescence or adulthood. In Norway, overweight or obesity in early childhood was reportedly associated with obesity in adolescence.^8^ Furthermore, in Germany, a population‐based study of about 50000 participants reported that most of the adolescents with normal weight had normal weight throughout their childhood.^9^ They also reported that obesity in adolescence was explained by rapid weight gain between ages 2 and 6 years.

Apart from the above studies, several previous studies also examined the relationship between birth weight or early childhood weight and obesity in later life, and most included subjects who were younger than the cohort in the present study at the time of outcome^10^, ^11^, ^12^, ^13^, ^14^, ^15^, ^16^ or were studies with short follow‐up periods.^17^, ^18^ Furthermore, only a few studies considered the effects of both birth weight and early childhood weight on obesity among adolescaents.^7^, ^8^, ^9^ Though previous studies suggested that parental obesity is associated with obesity in children, prenatal factors that may influence overweight and obesity in later life are not well understood among the Asian population.^19^ Therefore, the purpose of our study, which included over a thousand mother‐child pairs in a 15‐year follow‐up from birth to adolescence using infancy and school age health check‐up data in Japan, was to examine the association of overweight/obesity at age 15 years with birth weight, overweight/obesity in early childhood and prepregnancy overweight/obesity in mothers.

## 2 METHODS

### 2.1 Design and settings

This population‐based retrospective cohort study used the childhood and school health check‐up data collected by the Health, Clinic, and Education Information Evaluation Institute.^20^ In Japan, health check‐up is mandatory during childhood (at 1 month, 3 or 4 months, 6 or 7 months, 18 months and 3 years of age), and annually, for students at school. Children who were included in this study were born in Hofu City between April 2000 and March 2003 and received school health check‐up at 15 years of age. Hofu City is a regional city in the western part of the main island of Japan and had a population of approximately 116 000 in March 2019. The detailed Hofu study profile is reported elsewhere.^21^ In this study, inclusion criteria were children with available maternal pregnancy information, early health check‐ups (including birth information, and information at 18 months and 3 years of age) and school health check‐ups (at 15 years of age). Exclusion criteria were children with no information on at least one of the following: maternal pregnancy, early health check‐ups and school health check‐ups.

The study protocol was approved by the ethics committee of the Kyoto University Graduate School and Faculty of Medicine (R0852).

### 2.2 Measurements

Maternal pregnancy information was collected by self‐administrated questionnaires during early pregnancy. Demographic and lifestyle factors were provided by the mother and included maternal age at birth, height, body weight, job status, history of abortion, birth order of the infant, smoking and alcohol intake. In Japan, health check‐up for early childhood are conducted regularly from birth to 3 years of age to measure growth for the age of each child (at 1 month, 3 or 4 months, 6 or 7 months, 18 months and 3 years of age). In these visits, height and body weight were measured by health professionals, and data regarding children physical and mental development are collected. We included information on sex, birth order, birth weight, gestational age at birth, and height and body weight at 18 months and 3 years of age in our analysis. Annual school health check‐ups for 15‐year‐old students are performed once a year in June and includes physical examination conducted by health professionals.

### 2.3 Exposures

Exposure variables included birth weight and BMI and overweight/obesity at 18 months and 3 years. Birth weight was categorized according to the World Health Organization's definitions was as <2500 g (low birth weight) and ≥2500 g (normal birth weight). 22 The cutoff of ≥4000 g was considered for high birth weight based on previous evidence.^6^ Based on the International Obesity Task Force (IOTF)^23^ definition, early childhood overweight is defined as body mass index (BMI) >17.85 for boys and >17.64 for girls at 3 years of age. The IOTF criteria for overweight/obesity include children aged 2 to 18 years. Therefore, we only used BMI at 18 months as exposure for the analyses. BMI was calculated as weight (kg)/height^2^ (m^2^). Overweight/obesity in mothers was defined as BMI ≥25.

### 2.4 Outcomes

In our analysis, we considered both overweight and obesity as the outcome, because the number of children with obesity (BMI >28.32 in boys and >29.01 in girls) at age 15 years using IOTF criteria was very small. Hence, according to the IOTF criteria, boys with BMI >23.28 and girls with BMI >23.89 were considered as children with overweight/obese in our study.

### 2.5 Covariates

Among pregnancy information of mothers and data on children at birth, we included the following potential confounders in our analysis: gestational age of the child, birth order, mother's age group at pregnancy (<20, 20 to 35 and >35 years), prenatal smoking status (yes or no), and mother's working status (full time or other).

### 2.6 Statistical analysis

The children's baseline characteristics were summarized by birth weight (low or not) and overweight/obesity or normal weight, at age 3 years. Variables were reported either as mean and standard deviation or frequency and percentages. Generalized estimation equation (GEE) analyses were used to estimate the odds ratios (ORs) and 95% confidence intervals (CIs) of associations between prenatal and early childhood variables and overweight/obesity at age 15 years, because repeated measures were obtained for different ages.^24^ ORs for overweight/obesity at age 15 years were calculated with maternal prepregnancy BMI, overweight/obesity in mothers, birth weight (per 100 g), low/high birth weight, BMI at age 18 months and at age 3 years and overweight/obesity at age 18 months and at age 3 years. Data were also stratified by gender. We performed complete case analysis using GEE. All statistical analyses were performed using Stata software (version 13.0, StataCorp LP).

## 3 RESULTS

School health check‐up at 15 years of age was performed for 3082 students between June 2015 and June 2017. In 1581 (51.3%) children, who were followed‐up from birth till age 15 years, the information on their mothers' pregnancy were available. There were 788 (49.8%) boys and 793 (50.2%) girls. A total of 167 (10.6%) children were overweight/obese at age 15 years, of which 86 (10.9%) were boys and 81 (10.2%) were girls.

Table 1 describes the characteristics of the children and their mothers by low birth weight and overweight/obesity at age 3 years. Of 1518 children, 130 (8.2%) were born with low birth weight and 93 (5.9%) had overweight/obesity at age 3 years. Compared with normal birth weight children, more girls (58.5%) than boys (41.5%) had low birth weight, the mean gestational age was shorter in low birth weight children (36.3 weeks) than normal birth weight children (39.1 weeks), percentage of mothers aged over 35 years was higher in low birth weight children (8.5%) than normal birth weight children (7.5%) and mothers who smoked during pregnancy had more low birth weight children (12.5%) than normal birth weight children (10.5%). Compared with children who were not overweight/obese at age 3 years, more girls than boys (59.1% vs. 40.9%) were overweight/obese. The percentage of mothers aged over 35 years was higher in overweight/obese children (9.7%) than nonoverweight/obese children (7.4%). Mothers, who smoked during pregnancy, had a higher percentage of overweight/obese children (19.8%) than not overweight/obese children (9.9%).

**Table 1:** Mothers' and children's characteristics grouped by birth weight and overweight/obesity at age 3 years (N=1581)

		Birth weight			Weight at 3 years of age		
Characteristics	Total	Low birth weight (<2500 g)	Normal birth weight (≥2500 g)	*P*‐value	Normal	Overweight/obese	*P*‐value
Number of children	1,581	130	1397		1481	93	
Children's factors							
Boys	788 (49.8)	54 (41.5)	712 (51.0)	.040	746 (50.4)	38 (40.9)	.075
Girls	793 (50.2)	76 (58.5)	685 (49.0)		735 (49.6)	55 (59.1)	
Birth order^a^							
First birth	692 (43.8)	60 (46.5)	632 (45.4)	.705	646 (45.4)	40 (44.4)	.984
Second birth	564 (35.7)	44 (34.1)	520 (37.4)		530 (37.3)	34 (37.8)	
Third birth and more	264 (16.7)	25 (19.4)	239 (17.2)		247 (17.4)	16 (17.8)	
Birth weight (g)							
Mean (SD)	3037.9 (438.3)	2178.6 (392.8)	3117.8 (347.2)	<.001	3029.3 (435.5)	3175.8 (457.9)	.002
Birth weight^a^							
<2500 g	130 (8.2)	130 (100.0)	0 (0.0)	< .001	125 (8.4)	4 (4.3)	.171
2500‐3999 g (reference)	1376 (87.0)	0 (0.0)	1,376 (98.5)		1,288 (87.0)	82 (88.2)	
≥4000 g	21 (1.3)	0 (0.0)	21 (1.5)		17 (1.2)	4 (4.3)	
Gestational age^a^							
Mean (SD)	38.9 (1.6)	36.3 (2.8)	39.1 (1.3)	<.001	38.9 (1.6)	39.0 (1.7)	.135
<37 (preterm)	82 (5.2)	49 (37.7)	33 (2.4)	<.001	76 (5.2)	5 (5.6)	.892
BMI at age 18 months, mean (SD)^a^	15.9 (1.1)	15.7 (1.14)	16.0 (1.12)	.004	15.9 (1.10)	16.5 (1.13)	<.001
BMI at age 3 years, mean (SD)^a^	16.0 (1.1)	15.5 (1.23)	16.0 (1.12)	<.001	15.9 (1.07)	16.8 (1.34)	<.001
BMI at age 15 years, mean (SD)^a^	20.3 (3.0)	19.9 (3.5)	20.3 (2.9)	.178	20.1 (2.7)	23.4 (4.3)	<.001
Overweight/obese at age 15 years	167 (10.6)	18 (14.0)	147 (10.7)	.252	135 (9.2)	31 (33.7)	<.001
Maternal factors							
Mother's age at pregnancy							
<20 years	45 (2.9)	4 (3.1)	39 (2.8)	.907	42 (2.8)	3 (3.2)	.704
20–35 years	1417 (89.6)	115 (88.5)	1253 (89.7)		1329 (89.7)	81 (87.1)	
≥35 years	119 (7.5)	11 (8.5)	105 (7.5)		110 (7.4)	9 (9.7)	
Prepregnancy BMI, mean (SD)^a^	20.5 (2.8)	20.1 (3.0)	20.5 (2.7)	.066	20.4 (2.7)	21.6 (3.2)	<.001
Underweight	310 (19.6)	35 (26.9)	265 (19.0)	.090	299 (20.2)	10 (10.8)	.079
Overweight/obese	93 (5.9)	8 (6.2)	83 (5.9)	.202	81 (5.5)	12 (12.9)	.028
Having full‐time jobs	637 (40.3)	59 (45.4)	568 (40.7)	.295	588 (39.7)	44 (47.3)	.147
History of abortion	102 (6.5)	6 (4.6)	93 (6.7)	.366	101 (6.8)	1 (1.1)	.029
History of spontaneous abortion	228 (14.4)	19 (14.6)	206 (14.8)	.968	212 (14.3)	14 (15.1)	.844
Prenatal smoking^b^	160 (10.1)	16 (12.5)	140 (10.5)	.481	141 (9.9)	17 (19.8)	.004
Prenatal alcohol intake^b^	422 (26.7)	34 (27.0)	377 (28.4)	.742	387 (27.4)	31 (36.1)	.083

*Note*. Data are shown as number and percentage or mean and standard deviation.

Abbreviations: BMI: body mass index; SD: standard deviation.

a Numbers (percentages) of subjects with missing data were reported for the following variables: birth order, 61 (3.9%); birth weight, 54 (3.4%); gestational age, 28 (1.8%); BMI at age 18 months, 1 (0.06%); BMI at age 3 years, 7 (0.4%); BMI at age 15 years, 19 (1.2%); maternal prepregnancy BMI, 118 (7.5%); smoking status of mothers, 68 (4.3%); and alcohol intake of mothers, 76 (4.8%).

b Responses to smoking or alcohol intake statuses were yes, no, or sometimes.

Compared with the normal weight group, low birth weight children had higher percentages of overweight/obesity at age 15 years (14.0% vs. 10.7%). Similarly, compared with the normal weight group, children with overweight/obesity at age 3 years had higher percentages of overweight/obesity at age 15 years (33.7% vs. 9.2%).

Table 2 presents the results of GEE analyses for the association of overweight/obesity at age 15 years with birth weight, BMI at age 18 months and age 3 years and maternal prepregnancy BMI. Overall results for all subjects (N=1369) revealed no association of overweight/obesity at age 15 years with birth weight (adjusted odds ratio [aOR]: 0.97, 95% CI: 0.92–1.0, p=0.311) or BMI, per each 1 kg/m^2^ increase, at age 18 months (aOR: 0.87, 95% CI: 0.69–1.11, *P*=.273). BMI, per each 1 kg/m^2^ increase, at age 3 years was associated with higher risk of overweight/obesity at age 15 years (aOR: 2.15, 95% CI: 1.71–2.70, *P*<.001). Furthermore, maternal prepregnancy BMI, per each 1 kg/m^2^ increase, was associated with overweight/obesity at age 15 years (aOR: 1.17, 95% CI: 1.10–1.24, *P*<.001).

**Table 2:** Association of overweight/obesity at age 15 years with birth weight, body mass index, and maternal prepregnancy BMI

	Total (N=1369)		Boys (N=678)		Girls (N=691)	
Variables	Adjusted OR (95% CI)*	*P*‐value	Adjusted OR (95% CI)*	*P*‐value	Adjusted OR (95% CI)^a^	*P*‐value
Children's birth weight	0.97 (0.92‐1.03)	.311	0.95 (0.88‐1.03)	.219	0.99 (0.92‐1.08)	.879
(100 g increase)	1		1		1	
Children's BMI at age 18 months	0.87 (0.69‐1.11)	.273	0.76 (0.54‐1.07)	.118	1.04 (0.72‐1.49)	.847
(1 kg/m^2^ increase)	1		1		1	
Children's BMI at age 3 years	2.15 (1.71‐2.70)	<.001	2.40 (1.70‐3.40)	<.001	1.97 (1.44‐2.70)	<.001
(1 kg/m^2^ increase)	1		1		1	
Maternal prepregnancy BMI	1.17 (1.10‐1.24)	<.001	1.11 (1.02‐1.21)	.013	1.23 (1.13‐1.33)	<.001
(1 kg/m^2^ increase)	1		1		1	

Abbreviations: BMI: body mass index; CI: confidence interval; OR: odds ratio.

a Adjusted for gestational age, birth order, mother's age, prenatal smoking, and mother's working status.

Table 3 presents the results of the GEE analyses for the association of overweight/obesity at age 15 years with low/high birth weight, overweight/obesity at age 3 years and prepregnancy overweight/obesity in mothers. Overweight/obesity at age 3 years was associated with an increased risk of overweight/obesity at age 15 years overall (aOR: 4.26, 95% CI: 2.51–7.25, *P*<.001). This association was observed among girls (aOR: 6.48, 95% CI: 3.25–12.9, *P*<.001) but not among boys (aOR: 2.29, 95% CI: 0.92–5.69, *P*<.075). Similarly, prepregnancy overweight/obesity in mothers was associated with overweight/obesity at age 15 years for subjects (aOR: 2.46, 95% CI: 1.41–4.30, *P*=.002). This association was only observed among girls (aOR: 2.89, 95% CI: 1.30–6.41, *P*=.009) and not among boys (aOR: 2.18, 95% CI: 0.98–4.88, *P*=.057). On the other hand, no association of overweight/obesity at age 15 years with low birth weight (aOR: 1.54, 95% CI: 0.81–2.95, *P*=.188) or high birth weight (aOR: 2.06, 95% CI: 0.57–7.44, *P*=.272) was observed.

**Table 3:** Association of overweight/obesity at age 15 years with categorized birth weight, overweight/obesity at 3 years of age, and prepregnancy overweight/obesity in mothers

	Total (N=1370)		Boys (N=678)		Girls (N=692)	
Variables	Adjusted OR (95% CI)^a^	*P*‐value	Adjusted OR (95% CI)^a^	*P*‐value	Adjusted OR (95% CI)^a^	*P*‐value
Children's birth weight						
<2500 g	1.54 (0.81‐2.95)	.188	2.25 (0.90‐5.63)	.083	1.07 (0.42‐2.74)	.882
2500‐3999 g (reference)	1		1		1	
≥4000 g	2.06 (0.57‐7.44)	.272	1.40 (0.14‐14.4)	.779	2.52 (0.52‐12.3)	.253
Children's overweight/obesity at age 3 years	4.26 (2.51‐7.25)	<.001	2.29 (0.92‐5.69)	.075	6.48 (3.25‐12.9)	<.001
(Reference: no)	1		1		1	
Mothers' prepregnancy overweight/obesity	2.46 (1.41‐4.30)	.002	2.18 (0.98‐4.88)	.057	2.89 (1.30‐6.41)	.009
(Reference: no)	1		1		1	

Abbreviations: BMI: body mass index; CI: confidence interval; OR: odds ratio.

a Adjusted for gestational age, birth order, mothers' age, prenatal smoking, and mothers' working status.

**Figure 1:**
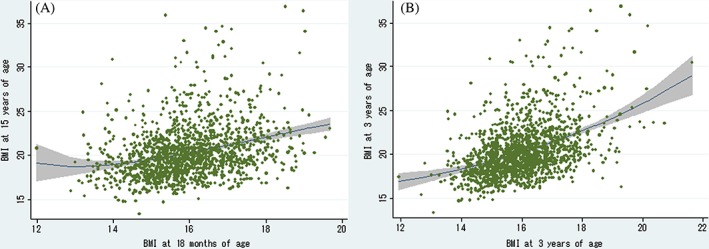
Plots of BMI at 18 months and three years of age with BMI at age 15 years. In the above graph, the two‐way linear prediction with 95% confidence interval of the mean is shown. The shaded area corresponds to the 95% confidence interval of the mean. BMI: body mass index

Figure 1 shows the plots of BMI at age 18 months and at age 3 years, with BMI at age 15 years. The slope of predicted regression line for BMI at age 3 years was steeper compared with that for BMI at 18 months and associated with overweight/obesity at age 15 years.

## 4 DISCUSSION

In this population‐based longitudinal study of Japanese adolescents, we found an association between overweight/obesity at 3 years with overweight/obesity at age 15 years. Furthermore, prepregnancy overweight/obesity in mothers was also a strong predictor of overweight/obesity at age 15 years among girls. We did not find any statistically significant association between birth weight and overweight/obesity at age 15 years.

Our study is in accordance with existing evidence showing an association between early childhood BMI and overweight/obesity at age 15 years. A large‐scale population‐based study in Germany showed that 90% of children who were obese at age 3 years remained overweight or obese in adolescence.^9^ The results of our study further supports the results of this study, and we observed that the association was strong with overweight/obesity at age 3 years. On the other hand, Evensen et al. reported that BMI in early childhood (2‐5 and 5‐7 years) is associated with overweight/obesity at 15–20 years of age and that the effect of overweight/obesity at 5–7 years of age is stronger than that in earlier life.^8^ This suggests that BMI in early childhood (around 5 to 7 years) may be more predictive of obesity in adolescence. However, the sample sizes of the previous study and the present one were not large enough to conclusively prove the association between obesity in early life and obesity in adolescence; therefore, further studies that include a longer follow‐up and larger sample size are needed.

Previous studies suggested that obesity among parents is associated with obesity in children.^11^, ^15^, ^16^, ^19^, ^25^ Among those reports, a large scaled meta‐analysis, using individual data of prospective birth cohort in Europe and North America, reported that BMI of mothers is an important predictor of obesity in adolescence, rather than other pregnancy complications such as prenatal diabetes.^25^ Additionally, one study in Norway reported that girls were at a higher risk of the effect of obesity in mothers; however, the age reported for their study outcome was 7 years.^16^ Our study further showed that overweight/obesity in mothers before pregnancy was also a strong predictor of overweight/obesity at age 15 years among girls only. Though it is reported that obesity‐related behaviours, such as eating behaviour and physical activity, are shared by children and their mothers,^26^ this may be explained by the evidence that girls are more influenced genetically by their mothers or by the environment, including the mothers' diet.^27^

Based on our results, birth weight was not a predictor of overweight/obesity at age 15 years. Despite OR of 1.54 for low birth weight and 2.06 for high birth weight we did not observe any significant associations between birthweight and obesity/overweight in adolescence. Our results are similar to a previous Danish study, which showed that compared with birth weight of 3000 to 3500 g, the risk of overweight between 6 and 13 years increased consistently with higher birth weight.^7^ Barker et al hypothesized that foetal undernutrition was associated with an increased likelihood of cardiovascular complications in adulthood,^28^ which is backed by some evidence.^29^, ^30^ However, the effect of birth weight on obesity in adolescence may be modest, whereas the influence of BMI in early childhood may be stronger.^8^, ^9^ In summary, low birth weight itself may not explain the obesity in adolescence, and weight gain in early childhood is more important but it could be modified.

In Japan, obesity during childhood has increased due to the westernization of diet and lifestyle, becoming one of the public health problems. However, Japanese population‐based data indicated that the mean birth weight in Japan has been declining gradually, contrary to the trend in developed countries. These are suggested because of mothers being underweight or having inadequate gestational weight gain.^31^ In year 2000, the mean birth weight was 3004 g in boys and 2960 g in girls according to the national data, which was about 200 g less than the national mean in 1980.^32^ Furthermore, Takemoto et al reported an increase in the prevalence of low birth weight infants from 4.5% in 1979 to 8.3% in 2010.^33^ On the other hand, the national prevalence for underweight women between 25 and 29 years of age was 23.7% between 1996 and 2000.^34^ Although based on the results of this present study, a direct association between low birth weight and increased obesity in adolescence may not have been established, attention should be focused on postnatal feeding. Parents' feeding practices are important for the nutrition and eating behaviour of their children,^35^ and early childhood may be a critical period for establishing eating behavior.^36^ Therefore, accurate instruction or intervention may be important in children under the age of 3 years, who might be at a risk for obesity.

### 4.1 Strengths and limitations

The strengths of this study include the population‐based design, long‐term follow‐up period of up to age 15 years, and the use of health check‐up data, which are less biased compared with data from self‐administered questionnaire surveys. Furthermore, our study included prenatal and prepregnancy maternal factors. Though several studies have evaluated the association between some factors in early childhood and obesity in adulthood,^7^, ^8^, ^9^ studies that include both prepregnancy or prenatal maternal variables and early childhood factors along with long‐term follow‐up of obesity in children are limited. Furthermore, this is the first study to examine obesity in adolescence using a population‐based cohort with long‐term follow‐up in an Asian population.

There are several limitations to our study. First, we used the cutoff values of the IOTF criteria to determine overweight/obesity index. Brann et al reported that the IOTF criteria were less sensitive compared with the WHO classification and the Swedish body mass index cutoffs,^37^ while Komiya et al showed these criteria to be more sensitive than the Rohrer index and BMI 95th percentile cutoff for BMI.^38^ The performance of IOTF criteria may depend on the references or ethnicity. If the IOTF criteria are more sensitive, but less specific than other obesity cutoff values, there might have been an overestimation of overweight/obesity in our study. Moreover, we only considered two‐time points (at 18 months and age 3 years) in measuring BMI. Our study did not consider adiposity rebound, where BMI begins to increase from its nadir around 6 years of age, and which has been reported to predict obesity in later life.^39^, ^40^ Furthermore, BMI itself might not be a suitable criterion in early childhood, a period characterized by rapid growth. The accuracy of other body fat indices such as youth triponderal mass index, which was introduced to estimate the fat level in young children, has not been confirmed.^41^, ^42^ Secondly, the study outcome was obesity at age 15 years only, and did not include diabetes or other cardiovascular outcomes in adulthood, which would have required a much longer follow‐up period. Further studies, with a longer follow‐up to include information on cardiovascular events in adulthood, are also needed. Thirdly, we did not fully adjust for all confounding factors that are associated with obesity in adolescence. In addition to diet and exercise, paternal factors and other environmental factors may be important in obesity in adolescence.^43^ Furthermore, though siblings share the genetic background and environment, and the effect of these factors among siblings should be adjusted for, we could not include sibling information in our analysis. Fourth, there may be selection bias in our study because the subjects were from one city in Japan and the follow‐up rate of our subjects was not high, which may lead to limited external validity. Furthermore, though our cohort included over a thousand children, our sample size may have been insufficient; thus, the generalizability of the results is unclear.

## 5 CONCLUSION

Our study showed associations between overweight/obesity at age 3 years and overweight/obesity at age 15 years. Furthermore, prepregnancy overweight/obesity in mothers was also a strong predictor of overweight/obesity at age 15 years among girls. Lifestyle factors, including eating habits, are not fully developed in early childhood and can be modified. It may be necessary to intervene on overweight/obesity in early childhood, to prevent obesity in adolescence.

## CONFLICT OF INTEREST

Koji Kawakami received research funds from Sumitomo Dainippon Pharma Co., Ltd.; Stella Pharma Corporation; CMIC Co., Ltd.; Suntory Beverage & Food Ltd.; Novartis Pharma K.K.; and Bayer Yakuhin Ltd. and consulting fees or speaker honoraria from Kyowa Hakko Kirin Co., Ltd.; Kaken Pharmaceutical Co., Ltd.; Astellas Pharma Inc.; Mitsubishi Tanabe Pharma Co.; AbbVie Inc.; Santen Pharmaceutical Co., Ltd.; Daiichi Sankyo Co., Ltd.; Takeda Pharmaceutical Co., Ltd.; and Boehringer Ingelheim Japan, Inc. and is a stockholder of the School Health Record Centre Co., Ltd. and Real World Data Co., Ltd. The other authors have no conflicts of interest to declare.
